# Magnetic tracking of eye position in freely behaving chickens

**DOI:** 10.3389/fnsys.2013.00091

**Published:** 2013-11-19

**Authors:** Jason S. Schwarz, Devarajan Sridharan, Eric I. Knudsen

**Affiliations:** Department of Neurobiology, Stanford University School of MedicineStanford, CA, USA

**Keywords:** eye tracking, bird vision, oculomotor control, eye movements, behavior, saccade, fixation, peck

## Abstract

Research on the visual system of non-primates, such as birds and rodents, is increasing. Evidence that neural responses can differ dramatically between head-immobilized and freely behaving animals underlines the importance of studying visual processing in ethologically relevant contexts. In order to systematically study visual responses in freely behaving animals, an unobtrusive system for monitoring eye-in-orbit position in real time is essential. We describe a novel system for monitoring eye position that utilizes a head-mounted magnetic displacement sensor coupled with an eye-implanted magnet. This system is small, lightweight, and offers high temporal and spatial resolution in real time. We use the system to demonstrate the stability of the eye and the stereotypy of eye position during two different behavioral tasks in chickens. This approach offers a viable alternative to search coil and optical eye tracking techniques for high resolution tracking of eye-in-orbit position in behaving animals.

## Introduction

Spatial vision research in non-primate species, such as birds and rodents, is becoming increasingly common (Harmening et al., 2011; Huberman and Niell, 2011; Sridharan et al., 2013; Starosta et al., 2013). The experiments typically involve taking measurements from animals that have their heads immobilized and are either passive (Longordo et al., 2013) or engaged in simple behaviors (Adesnik et al., 2012; Ayaz et al., 2013). There is growing evidence that neural activity in the head-immobilized condition can differ significantly from activity observed in freely moving animals (Ravassard et al., 2013). However, in order to measure the effects of visual stimuli at specific locations in the visual field, the positions of the eyes relative to the locations of stimuli must be known; monitoring eye positions accurately in freely moving animals has proven difficult. Here we describe a method for tracking eye-in-orbit positions that can be applied to freely moving animals, even in species with small heads.

A classical method for monitoring eye-in-orbit position is the electro-oculogram (EOG). The EOG detects the standing DC dipole of the eye with electrodes implanted in the orbit on either side of the eye. The magnitude of the recorded signal varies as the eye rotates. The advantage of EOG recordings is that they can be measured in freely behaving animals with minimal hardware attached to the head. The disadvantage is that the amplitude of the eye's dipole drifts with ambient illumination (Arden and Kelsey, 1962), and can change within tens of seconds, making EOG recordings unreliable for measuring absolute eye-in-orbit position.

Another frequently used method measures eye position with a search coil. The search coil method involves surgically implanting a coil of wire in the sclera around the eye and placing the animal at the center of an alternating, high-frequency electromagnetic field established by large induction coils that surround the animal (Fuchs and Robinson, 1966). The current induced in the eye coil depends on the angle of the coil (and thus the eye) relative to the field. The advantage of the search coil technique is that it provides high-resolution measurements (in both space and time) of the absolute eye-in-orbit position. The disadvantages are that the eye coil and its leads encumber the eye and the eye must remain within the volume of space where the magnetic field remains calibrated. Thus, it cannot be used in animals that are free to move. Also, the eye coil leads are fragile and repairing them requires additional surgeries.

A third method is optical tracking of eye position. This technique measures eye-in-orbit position by imaging physical landmarks in the eye and reflections of incident light sources from known locations (Kimmel et al., 2012; Wallace et al., 2013). The advantages of optical tracking are that no hardware needs to be attached to the eye and, with appropriate calibration, the absolute position of the eye-in-orbit can be derived from the images. The disadvantages are that real time tracking is expensive, computationally intensive requiring dedicated hardware, and the spatial and temporal resolution (particularly of head-mounted systems relevant for head free behaviors) tend to be low compared to that of the other techniques (typically < 50 Hz, Wallace et al., 2013; Yorzinski et al., 2013). In addition, the camera and incident light sources must be attached to the skull. Attaching such equipment to the skull without encumbering the animal or obstructing its visual field is difficult, especially for small animals such as birds and mice.

We report here a new technique that overcomes many of the weaknesses of these traditional techniques. This technique, called magnetic eye tracking, involves surgically implanting a small magnet under the temporal conjunctiva of the eye and attaching a magnetic displacement sensor to the skull. Both the magnet and the sensor can be small and lightweight. Magnetic eye tracking offers stable, reliable measures of absolute eye-in-orbit position, with high spatial and temporal resolution in real time and with equipment that can be carried easily by small, freely moving animals. Past studies have utilized Hall effect sensors for magnet eye tracking (Salas et al., 1999; Rodríguez et al., 2001; Schlageter et al., 2001; Kim et al., 2004). The technique we report here utilizes, instead, anisotropic magnetoresistive Wheatstone bridge elements, which offer spatial resolution and magnet-to-sensor gap performance that is superior to Hall effect sensors.

We developed this technique using the domestic chicken as our subject of study. The eye movements of chickens have been reported previously (Pratt, 1982). Eye saccades in birds involve the simultaneous execution of two different kinds of eye movement, each kind having a different purpose (Wallman and Pettigrew, 1985; Pettigrew et al., 1990; Yang et al., 2008): one is a step-shift in the orientation of the eye; the second is a rapid (15–30 Hz) oscillation of the eye. The eye oscillations are required because the avian retina contains no blood vessels, and cells of the inner retina depend on receiving oxygen and nutrients from a heavily vascularized structure (the “pecten oculus”) that protrudes from the optic nerve head. The oscillations of the eye disperse these substances from the pecten across the vitreous chamber to the inner retina.

This study reports on the performance of magnetic eye tracking in chickens that are engaged in goal directed behaviors. Chickens were trained to perform either a gaze-fixation task or a targeted pecking task. These tasks are typical of those used for quantitative research on visual perception and spatial attention, as they involve responses for reporting decisions that can be readily quantified in both space and time. In this study, we employ magnetic eye tracking to document the exceptional consistency of eye-in-orbit positions when chickens are engaged in these tasks.

Magnetic eye tracking will be of greatest utility in studies of small animals, such as birds and mice. However, it is also applicable to larger animals, such has non-human primates and even humans (if the implanted magnet were replaced by a magnetic contact lens). This technique overcomes shortcomings of both search coil-based and optical-based tracking systems for monitoring eye-in-orbit positions in freely behaving animals.

## Methods

Female white leghorn chickens (*Gallus gallus domesticus*) of the “Hyline” strain were used for all experiments. Data were acquired from four animals. Animals were housed individually, in visual and auditory contact with conspecifics, under a 14/10-h light/dark cycle. Water was provided *ad libitum*, but food was restricted to maintain individuals at 80% of their free-fed weight, and food was used as a reward in the behavioral tasks. All experiments were done in accordance with the guidelines of the Stanford Institutional Animal Care and Use Committee.

### Magnetic displacement sensor

**Figure 1:**
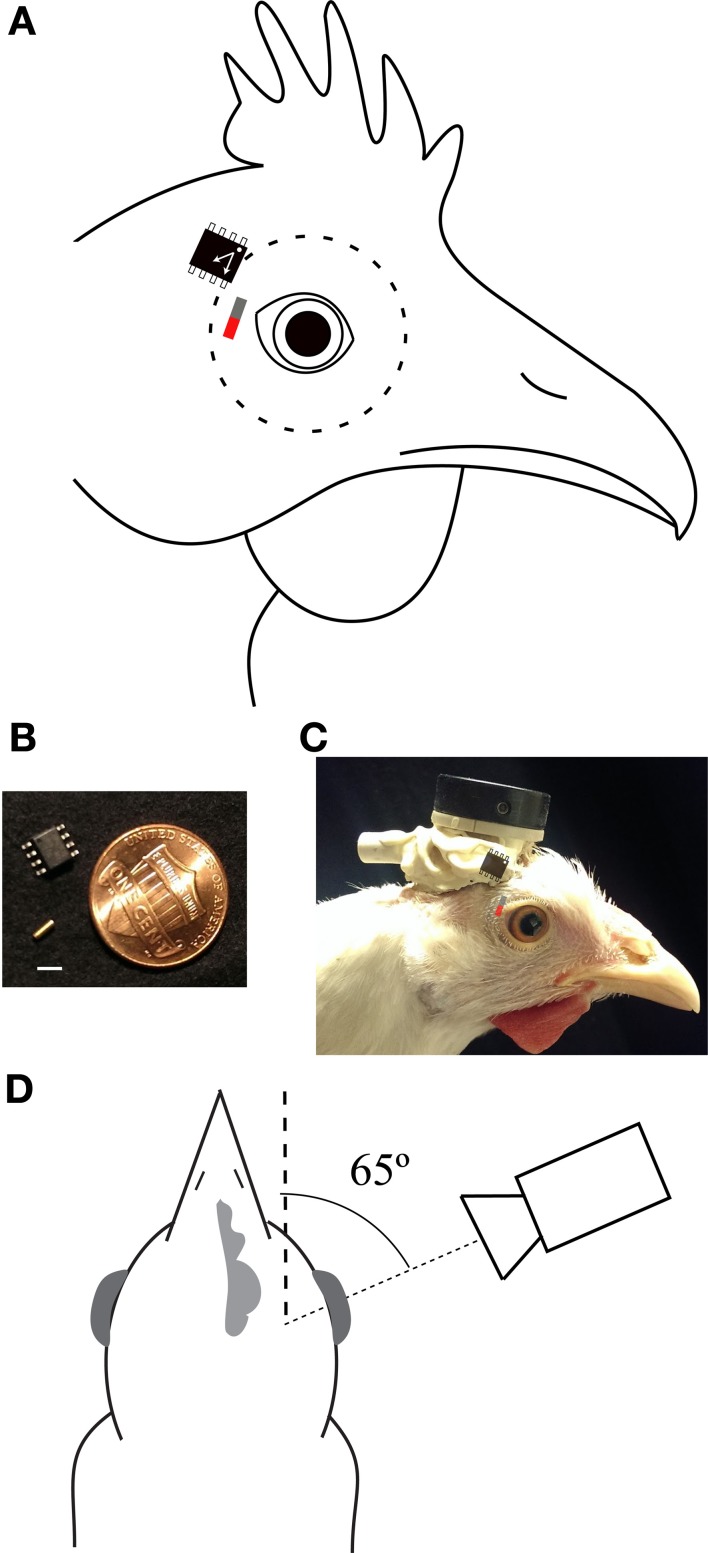
**Preparation. (A)** Optimal positions and orientations of sensor and magnet (red/gray bar). The dashed line indicates the extent of the eyeball. **(B)** The magnet (gold cylinder) and sensor in comparison to a U.S. penny. Scale bar is 3 mm. **(C)** Picture of a chicken with the magnetic tracking system implanted (neither is directly visible). The locations of the underlying magnet and sensor are overlaid using the same convention as in panel **(A)**. The head plate to which the sensor is attached and the Omnetics connector through which power is provided and sensor voltages recorded are apparent. **(D)** A schematic of the optical recording arrangement. The camera was aligned with the pupillary axis (fine dashed line), 65° lateral to the parasagittal plane of the head (coarse dashed line). Distances are not to scale.

The magnetic tracking system utilized magnetic displacement sensors to detect the positions of magnets implanted on the lateral surfaces of the eyes. The sensors were Honeywell HMC1512 2-axis magnetic displacement sensors (size: 4.8 × 5.8 mm; weight: 76 mg; Figure 1B), which contained a saturated-mode Wheatstone bridge sense element, the output voltage of which was modulated by the direction of magnetic flux passing over the sensor surface (HMC1512 Product datasheet: www51.honeywell.com/aero/common/documents/myaerospacecatalog- documents/Missiles-Munitions/HMC1501-1512.pdf). These sensors were designed to detect the direction of a saturating magnetic field and measure linear and angular displacements.(Honeywell magnetic sensors application notes: www51.honeywell.com/aero/common/documents/Applications-of-Magnetic-Position-Sensors.pdf). Honeywell documentation rates the displacement sensitivity of these sensors at <100 microns at a gap of 8 mm between the magnet and sensor. The sensor is designed to work with a “saturating” magnetic field of at least 80 Oe (or ~79.92 Gs, assuming the field is moving through tissue). If the magnetic field strength falls below this level, the sensor voltage output becomes a function of both magnet field direction and magnetic field strength. The HMC1512 sensor has two sensing elements offset by 45° in their directional sensitivity.

### Implantation of magnetic eye tracker

Surgeries were carried out while the birds were anesthetized with isoflurane (1.5%) in a mixture of oxygen and nitrous oxide (5:4). First, a headplate was cemented to the cranial bone on top of the head. Then, two magnetic sensors were cemented to the headplate, one adjacent to each eye (Figures 1A,C). Each sensor was positioned so that the long axis of the sensor chip was parallel to the axis of eye vergence (approximately horizontal during gaze fixation and pecking; Figure 1A). A small, cylindrical, gold-plated neodymium NeFeB magnet (size: 1 × 3 mm; weight: 17 mg; grade: N52; surface magnet flux: 900 Gs; axially magnetized along the 3 mm dimension; Figure 1B) was implanted under the temporal conjunctiva of each eye as follows: a hypodermic needle (25 G) was used to make a pocket under the conjunctiva; a larger needle (22 G) was used to expand the pocket; the magnet was placed in the bevel of an 18 G needle, the tip of which was used to expand the opening of the pocket. The magnet was then pressed into the pocket with non-magnetic forceps. The opening was sealed with cyanoacrylate adhesive (Vet Bond), irrigated with sterile saline, and medicated with sterile ophthalmic antibiotic ointment. Best results were achieved when the poles of the magnet were oriented perpendicularly to the axis of the sensor (Figure 1A; Discussion). We observed rare instances of bio-incompatibility. These appeared to be related to instances in which the gold plating on the magnet was disrupted, leading to formation of a black precipitate and local inflammation. Some animals had magnets implanted for over 7 months without issue.

The sensors were powered by a high capacity (7 Ah), 12 V lead acid battery connected directly to the Vbridge and ground leads of the sensors. Microconnectors (Omnetics) were used to couple power and recording leads to the sensors, and all leads were tethered by a wiring harness to the headplate. Output signals from the sensors were connected, without amplification, to a Tucker Davis Technologies RP2.1 real time processor for digitization (24-bit digitization, 105 dB S/N).

### Optical eye tracking

The absolute position of the eye-in-orbit was calibrated optically. The eyes of a chicken have clearly defined pupils, and we used an infrared (IR) human eye-tracker to monitor their positions with the head immobilized. The X-Y position of the pupil centroid in space was measured with an EyeTech TM3 optical eye tracker (sampling rate = 50 Hz) using custom MEX libraries developed to provide a MATLAB (v.2011a, The Mathworks Inc., Natick, MA) interface to vendor C API libraries (http://www.eyetechds.com/quick-link-2-version-2-5). Pupil center positions were recorded by the imaging system and were converted to degrees of rotation using geometry, as described previously for EOG calibration (Wohlschläger et al., 1993). First, pupil center positions were converted from pixel positions to millimeter positions using the camera field of view given the eye-camera distance, and these positions were converted to relative displacements by subtracting the median eye position for each dimension. Second, the relative displacements (ΔP) were converted to angles based on the assumption that the eye rotated about the midpoint of the pupillary axis, measured from the iris to be 4.58 ± 0.195 mm (standard error; *n* = 4 eyes from 2 birds). Hence, the eye rotation angle θ = *tan*^−1^ (ΔP/4.58).

### EOG recordings

EOG recordings were made using Ag/AgCl electrodes implanted subcutaneously at the temporal and nasal margins of the orbit. Differential signals were amplified using a custom low-impedance amplifier and digitized using an RP2.1 real time processor.

### Calibration of the magnetic sensor voltages

Magnetic sensor (and EOG) voltages were calibrated with the optical tracker while the animal's head was immobilized; the animal was otherwise awake, alert, and free to move its eyes. The implanted headplate was clamped to a jig that held the head in a stereotyped position. Recordings were made simultaneously from the optical tracker, magnetic tracker, and EOG electrodes for 2–4 min epochs as the animal executed frequent eye movements. To maximize the sensitivity of the optical tracker, the camera was aligned to the pupillary axis with the eye in its median position: approximately 65° lateral to the parasagittal plane, measured at the center of the head and at the same height as the eye (Figure 1D). Sensor voltages (and EOGs) were matched with optical tracker values recorded during periods of eye stability. These data were plotted and the conversion factor from voltages into angular degrees was based on a linear regression of these data. Magnetic sensor voltages were always calibrated immediately prior to a behavioral session.

### Head tracking

The position and orientation of the head were measured in all three spatial dimensions with an IR-based tracking system (V120 Duo, Natural Point). The system consisted of two IR cameras with fixed orientations mounted above the bird. IR markers were attached to the headplate. The tracking system provided measurements of absolute position in space relative to a zero point, and measured positions and angles with sub-millimeter and sub-degree spatial resolution, respectively, sampled at 120 Hz. Head position and orientation values, with 6° of freedom (X, Y, Z, yaw, pitch, roll), were stored. Beak direction, defined by the line passing through the midpoint between the eyes and the tip of the beak, was calculated using head position and orientation values. In the context of executing the behavioral tasks, the birds naturally tended to align the beak direction with visual targets on the screen, which we refer to as “gaze fixation.”

### Visual stimuli

The behavioral tasks are described in Results. Visual stimuli, consisting of a zeroing cross and a target (filled circle), were generated in MATLAB using the Psychophysics Toolbox extensions (Brainard, 1997), and were projected onto a calibrated viewing screen (Mitsubishi XD400U projector) for the gaze fixation task and displayed on a calibrated touch-screen (Elo Touchsystems Acoustic Pulse Recognition) for the pecking task (Sridharan et al., 2013).

## Results

### Head-immobilized eye movements

The chickens moved their eyes frequently when the head was immobilized. The movements consisted of saccadic shifts in eye position and rapid eye oscillations (15–30 Hz) that occurred during each shift. On average, these saccades occurred every 1.4 ± 0.75 s (mean ± standard deviation; *n* = 246 saccades; 6 eyes; 3 birds) and lasted 70 ± 36 ms, as measured with the magnetic tracker. During intersaccade intervals, the eye remained stationary.

**Figure 2:**
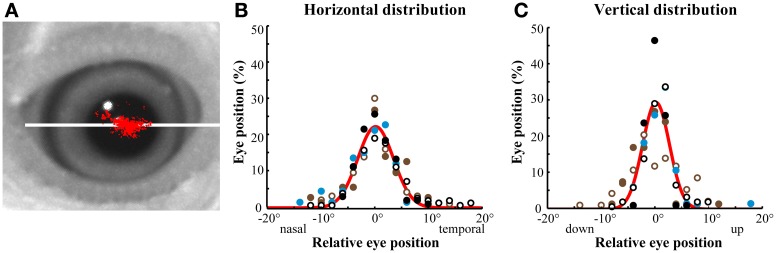
**Optically recorded eye position distributions. (A)** Representative distribution of eye positions in a head-immobilized bird during a single session measured with the optical eye tracker. Each red dot represents a sampled eye position. The distribution of eye movements was most extensive along the horizontal, vergence axis of the eye (white line). **(B,C)** Distributions of eye position in head-immobilized birds measured with the optical eye tracker. The eyes made small excursions from a median eye position. The range of eye motion was larger in the horizontal **(B)** than in the vertical **(C)** axis. Different colors represent different birds (*n* = 3). Open symbols represent left eye positions and solid symbols represent right eye positions. The bin size is 1°. The red line is a Gaussian fit of the mean in each bin across birds. In these and all subsequent plots, negative values represent nasal or ventral eye positions in the horizontal or vertical dimensions, respectively.

The optical tracker recorded the absolute eye-in-orbit X-Y positions during the intersaccadic intervals (Figure 2). The optical tracker was not sensitive to eye oscillations, cyclotorsion or movements along the Z-axis. Most of the detected variations in eye positions were along the axis of vergence, which was co-planar with the beak, referred to as the horizontal axis (Figure 2A). Although the eye rotated up to 40° along the horizontal axis and up to 30° along the vertical axis (perpendicular to the horizontal axis), the majority (61.26 ± 10.53%) of the variation in eye positions was distributed along the horizontal axis, and most of the time the eye remained at a stereotyped position within ±3° and ±2° of a median position along the horizontal and vertical axes, respectively (Figure 2).

**Figure 3:**
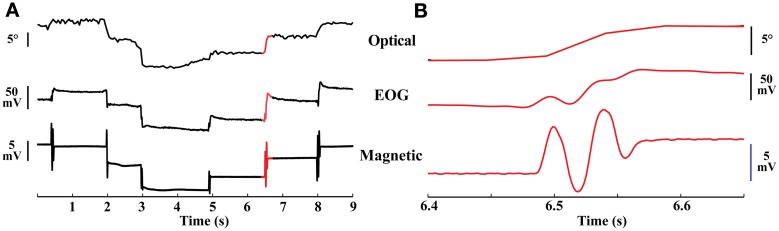
**Eye movements. (A)** Simultaneous traces of optical, EOG, and magnetic recordings of eye movements from a head-immobilized bird. Upper trace: optical recordings of eye position. Middle trace: EOG recordings. Lower trace: magnetic recordings. The magnetic trace had a high SNR, documenting the stability of the eye during the inter-saccade period. Traces sampled at 60, 610, and 610 Hz, respectively. Red highlighted region shown in higher resolution in **(B)**. **(B)** Higher magnification of the red highlighted region in **(A)** demonstrating the large amplitude of the eye oscillations.

The magnetic sensor and the EOG electrodes were positioned to be most sensitive to movements along the horizontal axis. Both the magnetic tracker and the EOG recordings resolved eye oscillations that were not detected by the optical tracker (Figure 3). Comparing the quality of the data from the three devices revealed the superiority of the magnetic tracker both in terms of temporal resolution and signal-to-noise. This superiority was particularly important for measuring the amplitude of eye oscillations and examining the fine temporal structure of eye movements (Figure 3B). In addition, the magnetic tracker (like the optical tracker) showed no evidence of the voltage drift that was apparent in EOG recordings during some intersaccade intervals (Figure 3A, intersaccade drift in middle trace).

**Figure 4:**
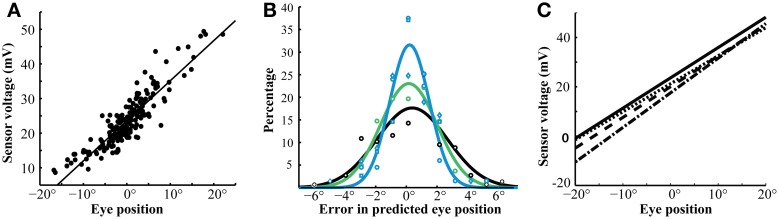
**Sensor calibration. (A)** Linear correlation of magnetic sensor voltage with optically derived eye position (*r*^2^ = 0.88, *p* < 0.001). This linearity extended over a wide range of eye positions. The data are from the left eye during a single session. **(B)** Differences between predicted eye positions, derived from calibrated magnetic sensor voltages, and optically recorded eye positions. The different colors are data from different birds. The error in the predictions was under 2° in greater than 75% of instances and under 1° in 47% of instances. Reliability of the predictions did not vary dramatically between sessions and only slightly between birds. The lines represent Gaussian fits for each bird. **(C)** Linear fits of eye position and magnetic sensor voltage from the same bird during four sessions. The relationship was stable throughout this 5-day period. Slopes: 1.228 (solid), 1.151 (dashed), 1.245 (dotted), 1.404 (dot-dash). Y-intercepts: 23.63 (solid), 21.52 (dashed), 20.04 (dotted), 17.56 (dot-dash).

Magnetic sensor voltages were compared with optically measured eye positions (θ, Methods) during intersaccade intervals when the eyes were stable (Figure 4A). Sensor voltages varied linearly with eye positions along the horizontal axis. These correlations were strong (mean *r*^2^ = 0.860 ± .066; *n* = 12 sessions; 4 birds) despite the influence that other axes of eye movement (vertical rotations detected by both tracking devices and cyclotorsion detected by the magnetic tracker, but not by the optical tracker) may have had on the measured values. When the linear regression was used to convert sensor voltages into eye positions along the horizontal axis, they corresponded with optical tracker values with an accuracy of < ± 2.5° for 92% of the measurements (Figure 4B). The relationship between sensor voltages and horizontal eye position drifted slightly across 5 days of measurements from the same eye (Figure 4C), probably due to slight changes in the position of the magnet under the conjunctiva. The mean of the absolute value of the error was 9.8, 17.8, and 34.4% on days 3, 4, and 5, respectively, when the calibration on Day 1 was applied to data collected on subsequent days. We calibrated the tracker signal daily to eliminate the effects of this drift.

#### Tracking eye movements in the head-unrestrained animal

Once the magnetic sensor voltages were calibrated with the animal's head immobilized, we used the magnetic tracker to follow eye-in-orbit movements in four chickens that were free to move their heads as they performed operantly conditioned tasks. One task required the bird to orient the beak toward a “zeroing cross” at the center of a videoscreen for 400 ms. During this period, the bird was required to maintain its beak direction (Methods) within ±8° of the cross and position the center of its head between 5 and 6 cm from the screen, as measured with the IR head tracker. After 400 ms of stable fixation, a target (3° bright dot) appeared 40° to the side, and the bird oriented the beak toward the target to receive a food reward.

**Figure 5:**
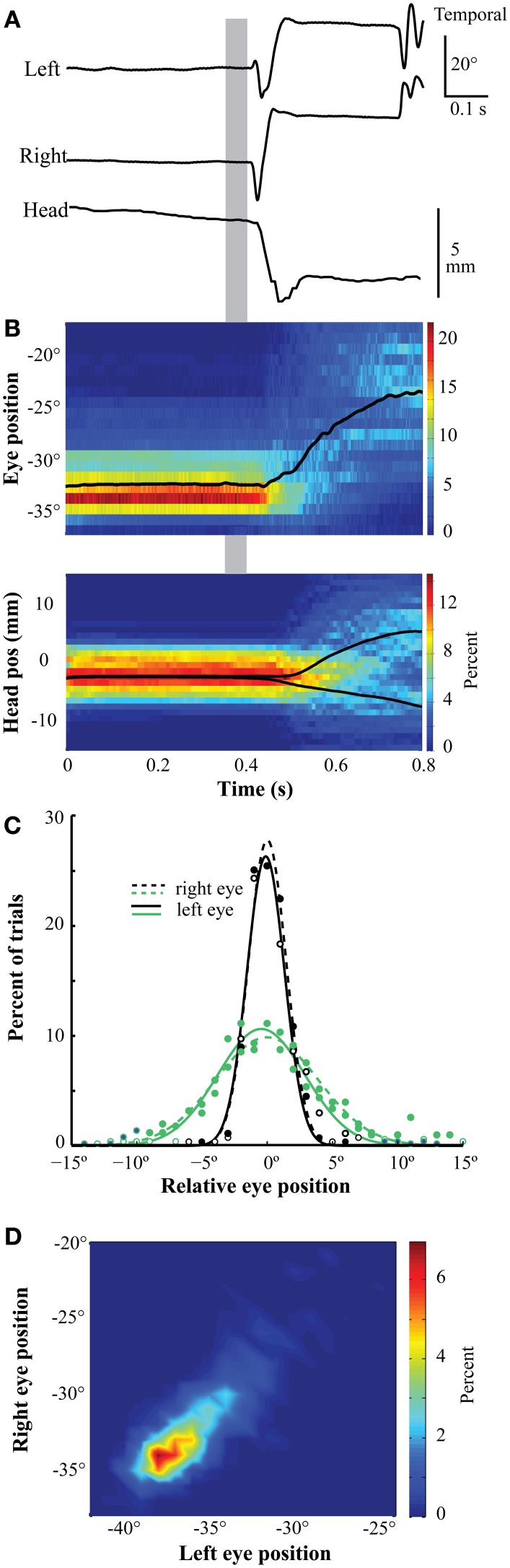
**Eye position during gaze fixation task. (A)** Representative traces of left eye, right eye, and head position during fixation and orientation. The positions are stable throughout the fixation period. Following stimulus presentation (gray bar), the eyes make a divergent saccade followed by a head orienting response to the left. **(B)** Eye position (upper panel) and head mediolateral position (lower panel) during a session were stable and stereotyped during fixation. At each time bin (0.61 ms for the eye and 8 ms for the head), the distribution of positions is represented with a heat map indicating the percentage of trials in which the eye or head was at that position (*n* = 657). The bin width is 1° for the eyes and 1 mm for the head. The black line represents mean position over time. The eye is in a converged position throughout the fixation period. In the head panel (below), the mean position was divided into trials in which the target was on the left or right, and the orientation responses of the head to the left or right can be observed. The gray bar indicates time of target presentation (0.35–0.39 s). **(C)** The distribution of eye positions during fixation was highly stereotyped across eyes and sessions. These distributions represent measurements made at the time of target onset (left border of gray bar). Positions are relative to the median position across trials in each session. Different colors represent distributions from different birds. Open symbols/solid lines are from the left eye and solid symbols/dashed lines are from the right eye. **(D)** The distribution of binocular eye positions is narrow and convergent. The measurements were made 300–425 ms following the initiation of fixation. The bin width was 0.05°.

Figure 5A shows representative traces of the left and right eye-in-orbit positions along the horizontal axis and head position along the mediolateral (left/right) axis during a single trial of the gaze fixation task. The traces begin with fixation of the zeroing cross and include the saccade to the target stimulus. Summaries of the distributions of eye and head movement traces as a function of time for an entire session (*n* = 657 trials) are shown in Figure 5B. While the beak remained stationary and oriented toward the zeroing cross, the eyes remained stationary in the orbits (Figures 5A,B). Eye positions were stereotyped across trials, as evidenced by the narrowness of the distribution in the time-position heat map (Figure 5B) and the histograms of eye positions at the time of target onset (Figure 5C). This stereotyped position was binocularly convergent, as demonstrated by the binocular position heat map at the time of target onset (Figure 5D). Approximately 100 ms after the target appeared (Figures 5A,B, gray bar), both eyes made temporally directed saccades to more diverged positions, as the bird fixated the more distant target. Shortly after the eye saccade began, the beak began to orient toward the target (Figures 5A,B). The same pattern of movements was observed in two birds.

**Figure 6:**
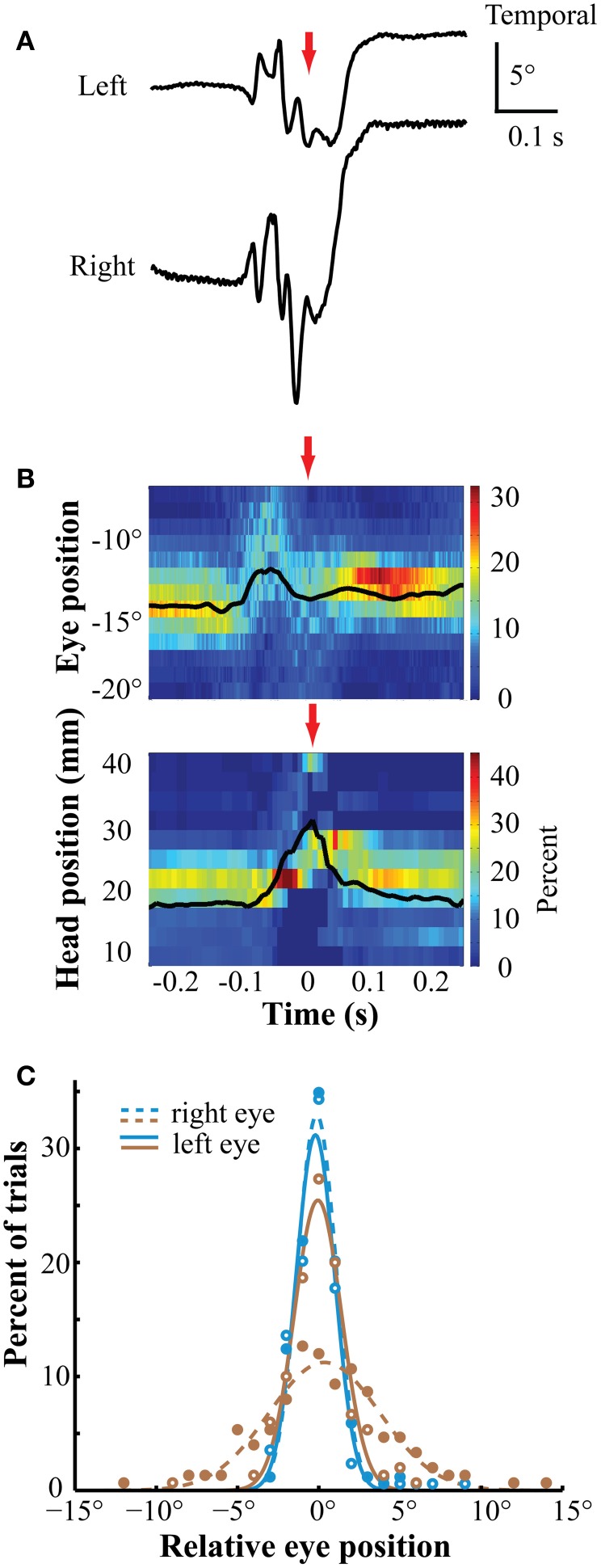
**Eye position during pecking task. (A)** Representative traces of left and right eye positions before, during and after the peck. The positions are stable both before and following the peck. During the peck, the eyes briefly diverged then converged. The red arrows indicate the timing of the peck **(A,B)**. **(B)** Eye position (upper panel) and head rostrocaudal position (lower panel) were stable and stereotyped during two fixation periods, one before and one after the peck. The bin width is 1° for the eyes and 3 mm for the head. The eye is in a nasal position throughout the period. Times on the abscissa are relative to the time of the peck. Colors represent percentage of trials in each position bin (*n* = 188). **(C)** The distribution of eye positions during the post-peck fixation was highly stereotyped across eyes and sessions. These distributions are taken 155 ms following the peck. Positions are relative to the median position across trials at the time of sampling in each session. Different colors represent distributions from different birds. Open markers/solid lines are from the left eye and solid markers/dashed lines are from the right eye.

A second task required the bird (*n* = 2) to orient the beak and peck two to eight times sequentially on a zeroing cross. Following this, a target (3° bright dot) appeared to one side and the bird oriented toward, and pecked on, the target to receive a food reward (for details, see Sridharan et al., 2013). Figure 6A shows representative traces of left and right eye positions immediately before and after a peck on the zeroing cross. Figure 6B summarizes the distributions of movement traces for one bird measured in one session (*n* = 188 pecks). For at least 150 ms preceding a peck on the zeroing cross (Figures 6A,B, red arrow), the eyes remained stationary in stereotyped positions, coincident with the period of head stability, consistent with the timing of pre-peck fixation reported previously (Goodale, 1983; Bloch et al., 1984; Macko and Hodos, 1985; Wohlschläger et al., 1993). During the ballistic peck motion, the eyes remained in a nasal position before, during, and following the time of impact, indicative of binocular convergence (Figure 6A). Following the peck, the eye-in-orbit positions were stable across time in highly stereotyped (Figure 6C) positions for a period of at least 150 ms (post-peck fixation, Goodale, 1983). The same pattern of movements was observed in both birds (Figure 6C).

## Discussion

We introduce a novel system for measuring eye movements in animals that are free to move their heads as they execute behavioral tasks. Similar patterns of eye movements have been reported previously in a variety of bird species measured with either EOGs or the search coil technique (Bloch et al., 1984; Pettigrew et al., 1990; Wohlschläger et al., 1993). Our magnetic system tracked both the step shift and the rapid eye oscillations associated with each eye saccade.

Two aspects of the eye movements reported here are noteworthy. First, the superior sensitivity of the magnetic eye tracker over EOG recordings revealed that the amplitude of the saccade oscillations are larger than has been previously reported (Pettigrew et al., 1990; Yang et al., 2008). Second, we demonstrate that when chickens fixate on a nearby visual stimulus, the head is stationary and the eyes assume a highly stereotyped, binocularly converged position. Such eye positions have been reported previously during natural pecking behavior in pigeons based on EOG recordings (Bloch et al., 1984; Wohlschläger et al., 1993). Our data show similar, stationary eye positions in tasks in which chickens either orient the beak toward a visual stimulus (Figure 5C) or peck on a visual stimulus (Figure 6C). The stability and stereotopy of the eye positions during these periods offer the opportunity to present visual stimuli at known locations in the visual field in studies of visual perception or spatial attention, even when only the orientation of the head is monitored.

The primary goal of this study, however, was to test a new method for monitoring absolute eye-in-orbit positions in freely moving animals. Magnetic eye tracking is of particular value for studies on small animals, such as birds and rodents, for which head-mounted optical systems are encumbering. It provides reliable measures of absolute eye positions, with high spatial and temporal resolution, and with minimal hardware attached to the head or eyes. Implantation of the magnets is invasive. However, it does not require attaching a wire lead to the eye, simplifying the procedure as compared to an eye coil implantation, and the absence of a wire lead avoids the need for its replacement due to damage. Moreover, although we used a wiring harness to power the sensors and to monitor their signals, these components could be battery-powered and wireless.

We tested various relative orientations of the sensor and magnet in order to determine the optimal arrangement for measuring horizontal eye movements. The quality of the signal from the magnetic sensor depended critically on the magnets operating in their linear range: the linear operating range provided the steepest gain (mV/degree) and, hence, the highest sensitivity to small changes in magnetic field orientation. Departures from this linear range significantly affected signal quality.

Departures from the linear range can arise from various sources. Signal quality depended critically on the relative orientations of, and distance between, the sensor and the magnet. There are several causes for this effect. One is that there is a specific range of magnetic field orientations over which the sensor voltages vary monotonically and linearly. Our calibration metric assumes a linear relationship between the sensor voltage and eye displacement. It requires, therefore, that the displacements of the eye do not shift the magnetic field orientation out of the range of linearity. Additionally, the sensor is designed to detect the direction of a saturating magnetic field (field strength of at least 80 Oe at the sensor) and large deviations of the eye could reduce the strength of the magnetic field to below saturating levels, thereby introducing a non-linearity to the relationship between sensor voltage and eye position. The magnetic sensors also detected cyclotorsions of the eye, which were not detected by the optical tracker; during initial testing, some placements of the sensor and magnet yielded weak correlations between horizontal eye position and sensor voltage. In such cases, the effects of eye cyclotorsion may have mixed with the effects of horizontal eye position. Finally, translational movements of the eye, particularly if they were in any way uncorrelated with the rotational movements, would have degraded the voltage-horizontal position relationship.

Despite these potential confounds, we consistently observed a robust linear relationship between horizontal eye position and sensor voltage. For studies requiring high-resolution eye-in-orbit measurements in both the vertical and horizontal dimensions, a second sensor could be mounted with sensing elements oriented orthogonally to those of the first sensor. The optimal arrangement of sensors and the magnet needs to be evaluated for each application. In addition, the calibration of sensor voltages changed slightly across days, possibly due to small changes in the position of the magnet or to changes in the supply voltage to the sensors. Therefore, to maintain maximum accuracy, the magnetic eye tracker must be calibrated at the beginning of each experimental session. We used a real-time optical eye tracking system to calibrate the magnetic tracking system. However, this calibration could be accomplished with a high-resolution camera and a pupil tracking algorithm implemented offline. If the system were applied to humans (contact lens-embedded magnet) or non-human primates, signal calibration could be performed behaviorally by inducing foveating eye saccades of known magnitude, as is done routinely to calibrate search coil signals or optical eye tracking. Unfortunately, this method of calibration is not an option for the vast majority of species that do not have foveas (such as chickens, mice, rats etc.) and, therefore, do not make precise foveating eye movements.

Most previous studies of visual processing or spatial attention have been conducted on animals that have had the head immobilized. This approach has been driven, in large part, by the need to present visual stimuli reliably at specific locations in the visual field. With the growing interest in studying visual responses in freely behaving animals, there is an increasing need for techniques that permit the reliable monitoring of real time eye position in head unrestrained animals. The magnetic eye tracker accomplishes this goal by providing accurate information about eye-in-orbit position with high temporal resolution, using lightweight, low profile hardware. In conjunction with an optical head-tracking system, this new method for unobtrusively tracking eye position enables studies in freely moving animals performing complex natural or trained behaviors, allowing for rigorous investigation of more nuanced, ethologically relevant problems.

### Conflict of interest statement

The authors declare that the research was conducted in the absence of any commercial or financial relationships that could be construed as a potential conflict of interest.
